# A qualitative formative evaluation of a patient facing intervention to improve care transitions for older people moving from hospital to home

**DOI:** 10.1111/hex.13560

**Published:** 2022-09-03

**Authors:** Rosie Shannon, Ruth Baxter, Natasha Hardicre, Thomas Mills, Jenni Murray, Rebecca Lawton, Jane K. O'Hara

**Affiliations:** ^1^ Yorkshire Quality and Safety Research Group, Bradford Institute for Health Research, Temple Bank House Bradford Royal Infirmary Bradford UK; ^2^ School of Psychology University of Leeds Leeds UK; ^3^ School of Healthcare University of Leeds, Baines Wing Leeds UK

**Keywords:** elderly care, evaluation, hospital discharge, patient involvement, patient safety, resilience engineering, transitional care

## Abstract

**Background:**

The Partners at Care Transitions (PACTs) intervention was developed to support older people's involvement in hospital to improve outcomes at home. A booklet, question card, record sheet, induction leaflet, and patient‐friendly discharge letter support patients to be more involved in their health and wellbeing, medications, activities of daily living and post‐discharge care. We aimed to assess intervention acceptability, identify implementation tools, and further develop the intervention.

**Methods:**

This was a qualitative formative evaluation involving three wards from one hospital. We recruited 25 patients aged 75 years and older. Ward staff supported intervention delivery. Data were collected in wards and patients' homes, through semi‐structured interviews, observation, and documentary analysis. Data were analysed inductively and iteratively with findings sorted according to the research aims.

**Results:**

Patients and staff felt there was a need for, and understood the purpose of, the PACT intervention. Most patients read the booklet but other components were variably used. Implementation challenges included time, awareness, and balancing intervention benefits against risks. Changes to the intervention and implementation included clarifying the booklet's messages, simplifying the discharge letter to reduce staff burden, and using prompts and handouts to promote awareness.

**Conclusion:**

The PACT intervention offers a promising new way to improve care transitions for older people by supporting patient involvement in their care. After further development of the intervention and implementation package, it will undergo further testing.

**Patient or Public Contribution:**

This study regularly consulted a panel representing the local patient community, who supported the development of this intervention and its implementation.

## 1 INTRODUCTION

### 1.1 Background and rationale

Emergency hospital readmissions are more common in patients aged 75 years and older,^1^ and as many as one in five people experience an adverse event post‐discharge.^2^ Up to one third of these readmissions and adverse events are potentially preventable.^3^, ^4^ An increase in long‐term conditions and reduced lengths of stay^5^ mean that older people often leave the hospital with ongoing and complex care needs. Missing referrals, errors with medication and equipment provision and poor communication are common problems that are reported by patients and families^6^, ^7^, ^8^, ^9^, ^10^, ^11^ and these may be indicative of poor quality discharges. Patients may therefore experience discharge with limited notice and have a poor understanding of their diagnosis or post‐discharge care.^6^, ^7^, ^8^, ^9^, ^10^, ^11^ The discharge period can be especially risky for people who may be deconditioned and still recovering,^12^ and yet are required to resume self‐care activities at home. Discharge is therefore a *stage* in a transition of care not an end‐point of care. Evidence on the effectiveness of transition interventions in reducing hospital readmissions is mixed.^13^, ^14^, ^15^, ^16^, ^17^, ^18^, ^20^, ^21^ Further, clarity about the active ingredients of such interventions is challenging to decipher because of the multiple and variable components that can be included.^13^, ^14^ Interventions can span across, or be restricted to, the pre‐discharge period (e.g., patient education, medicines reconciliation and/or predischarge planning), the bridging period (e.g., transitions co‐ordinator and/or patient‐centred discharge instructions) or the post‐discharge period (e.g., follow‐up and/or patient hotlines) periods.^13^, ^14^, ^15^, ^16^, ^17^, ^18^, ^19^, ^20^ However, there is some evidence that patient education with or without self‐management may contribute to reduced readmissions.^21^ More precisely, interventions that seek to ‘enhance patient capacity to reliably access and enact post‐discharge care’^21^ may contribute to better patient outcomes. The exact mechanisms for this, however, remain unclear.

#### 1.1.1 The rationale for patient and family involvement

Transitions involve knowledge transfer between multiple professionals, services and individuals.^19^ These transitions have been described by some authors as structural safety ‘gaps’, which heighten the potential for failures in communication and other safety failures.^22^, ^23^ Patients and their carers are the only constant throughout these transitions and so have opportunities to limit the impact of these safety gaps and support the safety of their care, by identifying or preventing mistakes through coordination across settings.^24^ However, opportunities to help close these gaps at transition depend on taking a more active role in their care in the hospital. Taking an active role is not without challenges, however, as patients can often ‘passively’ receive care and avoid asking staff questions.^25^, ^26^ Further, busy staff may not prioritize engaging in conversation with patients,^25^, ^26^ meaning that patients can sometimes return home ill‐prepared to manage themselves, their care, and condition. If the potential benefits for patients and families in ‘plugging the safety gaps’ across transitional care are to be realized, we need ways to support patients to be more active, recognized, and supported partners in their care during the hospital stay.

### 1.2 Partners at Care Transitions​​​​​​ intervention

A full description of the intervention development is presented elsewhere^27^ but summarized briefly here. To develop an intervention to support greater in‐hospital patient engagement in their care, we first explored the experiences and perspectives of healthcare professionals and older patients^26^, ^28^ and brought these together using Functional Resonance Analysis Method^29^ to model transitions of care from hospital admission to 30 days post‐discharge.^30^ This approach enabled us to identify four key activities that patients and families are responsible for (to varying degrees) following discharge from the hospital:

1. Understanding and managing their health and well‐being;
2. Understanding medications and how to manage them;
3. Managing activities of daily living;
4. Understanding what to expect after discharge and how to escalate care if needed.

In our resulting underpinning theory of change,^30^ we hypothesized that supporting patients to ‘know more’ and ‘do more’ in the hospital would better prepare them for being at home, thus improving transitional care outcomes. The Partners at Care Transitions (PACT) prototype intervention was co‐designed through a series of stakeholder workshops (patients, carers, healthcare staff and design team) and through the consolidation of evidence from a range of sources.^27^

The prototype PACT intervention comprises the following core patient‐facing components:

1. *An information booklet* for patients, encouraging them to be more involved in their care by retaining independence, signposting and offering suggested questions;
2. *A stand‐up ‘question card’* for patients to write and display their questions to staff, and promote communication between staff, patients, and families;
3. *A ‘hospital record sheet’* for patients to record events or conversations regarding their health;
4. *A ward induction leaflet* to orientate patients to ward routines and reduce the disorientation that can happen on hospital admission;
5. *A patient‐friendly discharge letter* based on the four functions, for staff to complete and provide to patients at discharge.

These materials were combined within a purpose‐designed envelope and provided to patients shortly after admission to the ward. The patient‐friendly discharge letter was given at discharge. The intervention was designed to allow flexibility and local adaptation in methods of supporting patients to undertake the activities, which is in keeping with emergent complex intervention development approaches,^31^, ^32^, ^33^, ^34^ for example, supporting patients to dispense their own medicine or having conversations to understand the purpose of their medications

#### 1.2.1 Research aims

In line with Medical Research Council guidance^35^ and more recent framework guidance^36^ that recommends iteration during complex intervention development, this study aimed to explore the acceptability of the prototype intervention and identify areas of improvement. We further aimed to identify implementation strategies. Our research aims were threefold:

1. Explore the acceptability and usability of the intervention for patients, caregivers and staff. Within the framework of acceptability, our study aims fit most closely with the constructs of intervention coherence (user understanding of the intervention), affective attitude (user feelings about the intervention) and burden (perceived effort required to use the intervention);^37^
2. Identify implementation strategies for the intervention;
3. Identify modifiable areas for improvement in relation to usability, usefulness and acceptability of the intervention.

## 2 METHODS

### 2.1 Study design and implementation

We conducted a formative evaluation using multiple qualitative methods, to explore the acceptability of the prototype PACT intervention and its initial implementation. We focused on gathering user perspectives (in‐patient older adults, carers, ward‐based practitioners) whose views were crucial in this developmental and early implementation phase.

We designed this formative study to have two phases. In the first phase, shortly after consent was provided, researchers introduced patients (*n* = 9) to the intervention, including an explanation of what it is and how to use it. This phase was designed to explore how best to deliver the introduction and to use this understanding to develop materials for staff to support them to undertake this introduction. This learning was then formatively integrated into the second phase, wherein the ward staff undertook the introduction of the intervention to patients (*n* = 16). Short multidisciplinary ward team briefing sessions were provided throughout the study to maintain staff‐level awareness and promote opportunities for them to support the intervention. These sessions also facilitated the development of staff‐facing implementation tools.

The study was given ethical approval by the North West—Greater Manchester East Research Ethics Committee (ref 18/NW/0636).

### 2.2 Setting and sample

We approached patients and staff on three wards (two older adult, one urology) in one hospital in Northern England, between December 2018 and April 2019. These specialties were purposively selected as they have a high proportion of older patients and provide a mix of acute and chronic care, which can result in different transitional challenges. We planned to recruit 25 patients and approximately 15 ward staff and carers where relevant.

Eligible patients were: aged 75 or over; likely to return to their own home; English‐speaking and; an in‐patient for at least one night. Patients were excluded if: they resided over 30 miles radius of the hospital; were at the end of their life; or were unable to give informed consent. Carers or relatives were also invited to participate in the study alongside the patient. Ward staff were purposively selected for interviews based on their involvement with the intervention. Interviewees were provided with verbal and written information and sufficient time to ask questions, before their providing written informed consent. Observations were conducted with verbal information and consent.

### 2.3 Data collection

Data were collected through three complementary methods—short semi‐structured interviews, observation of care and intervention use and examining the physical intervention components—by researchers experienced in these methods, patient groups and the background intervention development (R. S., T. M., N. H.). All data collection methods (including loosely structured topic guides) were informed by the Capabilities, Opportunities, Motivation‐ Behaviours (COM‐B) (model of behaviour change.^38^ This model aims to support intervention and implementation development by identifying appropriate techniques for targeted behaviours. In this study, we focused on the core components of the model (i.e., capability, motivation, and opportunity) that influence our initial targeted behaviour of interacting with the intervention. Data collection occurred within three specific settings: mainly across the selected wards, in participants' homes and on one occasion intermediate care settings. This design allowed us to ‘follow’ patients and their use of the intervention as they transitioned from hospital to home. All data, including reviewed documents, interviews (which were audio‐recorded where possible) and observations were documented on a semi‐structured ‘contact form’ after each data collection contact—(prompts included intervention use; feedback; suggested changes).

#### 2.3.1 Interviews—Patients and carers

With consent, and before being introduced to the intervention, we conducted interviews with patients to explore their current and desired involvement in their care, so we could later understand their intervention use in context. A second patient interview was conducted 7–14 days post‐discharge from the hospital to explore barriers to intervention use, how it was used and areas for improvement (usability, readability, content, aesthetics). See Supporting Information: Files S1 (outline of patient data collection), S2 (baseline interview topic guide for patients) and S3 (follow‐up interview topic guide for patients).

#### 2.3.2 Interviews—Staff

The guide (see Supporting Information: File S4) for staff interviews was similar to the patient guide, being informed by the COM‐B model of behaviour change.^38^ Interviews further explored staff views on the intervention, including areas for improvement and ideas for developing implementation tools. Interviews were conducted with a range of multidisciplinary ward staff who engaged with the intervention.

#### 2.3.3 Observation

Patient‐ and ward‐level observation was used to explore how patients, carers and staff interacted with the intervention. We undertook observation of a number of critical points in the process of staff delivering and patients receiving the intervention. These points included the introduction of the intervention to patients by staff, routine care interactions (e.g., ward rounds, dispensing medicines), visiting times and discharge. Short conversations with patients or staff involved in these observations helped contextualize what was observed, how the intervention could help staff and how they could use it in their role.

#### 2.3.4 Physical intervention components

On visits to patients' homes for post‐discharge interviews, we looked at and collected data about the intervention components given to patients, to explore how they had been used.

### 2.4 Data analysis

Given the practical orientation of our overall research aim, template analysis^39^ was used to orient the analysis to the three research questions. In keeping with a formative evaluation approach, data analysis was iterative, starting during data collection, with findings used to inform subsequent data collection. The main unit of analysis was the contact form (see Supporting Information: File S5). Each form was reviewed independently by one of three researchers (T. M., N. H., R. S.), annotating key data. These key data were then grouped by research aim, then sometimes split or moved following discussion, and developed into draft findings. This process was repeated across the study period, with emergent findings discussed at regular meetings with the wider team (including the programme manager, CI and research nurse staff) contributing to the process and final findings. Disagreements were resolved via consensus discussion.

There were two additional steps specific to research aims 2 and 3. To address the second research aim (development of potential implementation tools), the team first listed all the desired behaviours of both ward staff and patients/carers. These included delivering the intervention (e.g., staff giving it to patients), identifying the barriers to this behaviour (e.g., not perceiving the booklet to be useful to patients) and creating suitable responses to each barrier. We did this by systematically thinking through staff and patients' capacity, opportunity and motivation^38^ for intervention engagement (e.g., training to understand why the intervention is important and how to verbalize this to patients). To address the third research aim (improvement of the intervention components), we used a research design technique that allows intervention developers to concisely convey the importance of the intervention and merge it with the core message to share with users. This method involves condensing the information into four levels (five words; one sentence; one paragraph; two paragraphs). We applied this to the overarching aim stated within the booklet and to the four functional activities.

### 2.5 Patient and public involvement and engagement

A panel of individuals (and their carers) who were aged 75 years and older and who had recent experience of hospital transitions, supported prototype intervention and initial implementation strategy development (by attending codesign workshops), aided in the understanding of patient experiences throughout the research and contributed to the intervention improvements and development of the implementation strategies through monthly meetings with the researchers. They were instrumental in ensuring that the language used within the intervention materials was accessible, engaging, and impactful.

## 3 FINDINGS

We approached 57 patients and 25 consented to take part, along with 6 carers (Table 1). The primary reasons for declining participation were illness and disinterest in the research. During the study, two participants died and were withdrawn from the study. Recruited patients each had an average of four contacts (range: 1–8). Twenty‐five patients were interviewed at the point of consent, with 16 completing the post‐discharge interview. Interviews were conducted with 15 ward staff, most of whom had been involved in using the intervention, including a Consultant Geriatrician, an FY2 Junior Doctor, Nurses (*n* = 7), Healthcare Assistants (*n* = 4); Physiotherapists (*n* = 1) and Pharmacy Technician (*n* = 1). Seven were recorded and lasted an average of 25 min (range: 14–50), eight took place within the busy clinical environment and were not recorded. Observations took place over a 3‐month period (December 2018 to March 2019). These were conducted on all three wards, with the researchers visiting each ward several times per week, at different times of the day with each observation lasting up to 3 hours . There were 20 visits to conduct ward‐level observations, this provided 10 cases of observing passport use (booklet or discharge letter) and 8 cases of observing usual ward routines where the intervention may be discussed or used (e.g., handover meetings, medication rounds).

**Table 1:** Patient characteristics

Patient participants (*n* = 25)			
Age—median			84 (range: 75–99)
Gender—male			40% (*n* = 10)
Ethnicity—White			100% (*n* = 25)
Patient participants who had a carer recruited into the study			20% (6 carers for 5 patients)
Reason for admission			
Infection (sepsis, pneumonia, cellulitis, other)			40% (*n* = 10)
Fall or fractured neck of femur			28% (*n* = 7)
Other (incl. short of breath, pain, heart attack, other)			32% (*n* = 8)
Ward recruited from			
Urology			16% (*n* = 4)
Elderly Care 1			32% (*n* = 8)
Elderly Care 2			52% (*n* = 13)
Lives alone			40% (*n* = 10)
Intervention introduced to patient by			
Researcher			36% (*n* = 9)
Ward staff (healthcare or nurse)			56% (*n* = 14)
Did not receive			8% (*n* = 2)

Our analysis was focused on the three research questions of acceptability, potential implementation strategies and modifiable areas for improvement. We present the results of our analysis below, with reference to each research question in turn.

### 3.1 Research aim 1: Explore patient, caregiver, and staff acceptability and usability of the intervention

Most patients or their families read the booklet and a minority wrote in the booklet or question card, while the ‘hospital record sheet’ was not used. Reasons why patients and families did or did not appear to demonstrably using the intervention, included: the number of physical components; patient capacity or willingness to engage (either with their care or with the intervention); physical and mental health status (e.g., feeling unwell or unable to do more); not being ‘a reader’; feeling it might be helpful for ‘other’ patients, and a preference to look at the intervention at home. Where families engaged, this was often as a replacement for the patients (e.g., if the patient was unwell). The patients and carers who used it most were already actively involved in their care, the intervention therefore having a greater ‘fit’ with their usual behaviour. While there was a general consensus among staff, patients, and families that patients can and should be more involved in their hospital care, the booklet did not encourage this strongly enough.

Many found the question card difficult to write on and found it too ‘strong’ or formal an action to take to engage with staff. Patients would often reveal their uncertainties to researchers, but did not seem to view these as specific ‘questions’ for ward staff. Some suggested providing questions, rather than having a blank space. The question card was, however, used in positive unanticipated ways, such as communicating between different visiting relatives and staff writing the patient's question for the doctor.

*The patient's daughter remembers the booklet as being about getting home, doesn't know where it is now, ‘she's been very poorly so it was the last thing on her mind’. Daughter says they wrote a list of what they thought she was going to need and handed it to staff, the booklet ‘put us in mind of doing things like that’. Patient says she doesn't think she wrote anything down, wasn't in a state to be thinking of writing. They both see the Question card as a personal reminder to yourself*. (Researcher field note, conversation with patient and daughter, hospital visit)

Staff concurred that safe discharge of patients was challenging and saw the benefits of the intervention for staff and patients. Perceived benefits included streamlining and improving day‐to‐day and postdischarge communication. There were concerns that increasing patient demands or questions would increase staff burden. Staff interaction mainly occurred at the time of giving the intervention to patients. Infrequently, other staff interactions were observed, including a Therapy Assistant encouraging a patient to ask questions and a Junior Doctor discussing the intervention booklet with a patient. The ward induction leaflet was found to be useful to staff, with all three wards adopting it into their own routines by the end of the study.

*She thinks [the intervention] is a great idea. Says it helped them think differently sometimes, about patient interactions and why you're doing what you're doing, not just what you're doing… She says giving the intervention to patients was a particularly positive experience for one healthcare assistant, who can be negative but he's enjoyed being part of it and its given him a useful role and responsibility*. (Researcher field note, interview with Senior Nurses)

The patient‐friendly discharge letter was well received in principle by both patients and staff. However, we found that it was rarely completed by staff because of the lack of IT integration, the requirement for multidisciplinary input, and the potential to duplicate work. Furthermore, it required staff to provide written information in a lay‐friendly manner, about things that can be changeable or unclear (e.g., escalating symptoms, future appointments from services that they cannot control). Despite these challenges, the completed patient‐friendly discharge letter was received very positively by the few patients who received it.

*The patient friendly discharge letter was the first thing he mentioned when I arrived, ‘I got your letter!…it says exactly what happened, it kept us to date on what they've done, what they haven't done, who's going to do what’. His wife has also read it, he hasn't shown it to any other healthcare professionals. He is unsure about the letters description of his reason for admission, and might raise this with his GP*. (Researcher field note, Patient home visit)

Some staff reported that these factors collectively increased feelings of uncertainty regarding accountability and exposure to litigation.

*You can never cover every complication, especially with older people they don't present typically…ongoing tiredness could be an indication that they're going into renal failure or something like that, illness starts with some vague symptoms and it's trying to work out how not to falsely reassure people*. (Consultant interview)

### 3.2 Research aim 2: Identify implementation strategies for the intervention

Researchers used their experience and learning about introducing the intervention to patients to develop a loose guide for staff. This took the forms of small (A7, bullet points) and longer (A4, suggested script) laminated cards. The smaller version was most well‐received by staff. Patient engagement with the intervention appeared to increase with ward staff introductions suggesting greater perceived credibility. While many staff found introducing the intervention easy, some found the rationale difficult to explain or process. Nurses often lacked time to introduce the intervention and suggested the need to engage the wider multidisciplinary team.

Staff willingness and ability to engage with the intervention varied from enthusiasm to disinterest or avoidance. While team briefings effectively conveyed the key points of the intervention to staff, momentum was lost due to changing staff and daily pressures.

Among other challenges, staff found it difficult to balance intervention benefits against risks, for example, supporting patients to practise taking their medicines versus potential medication error risk or practising walking to maintain independence versus potential fall risk. This impacted the delivery of the flexible aspects of the intervention.

*[The Physiotherapist] generally likes the idea but had not seen anyone using it and felt that there was a lot of awareness raising work to be done before expecting staff to engage with it. ‘It's about knowing what we are meant to be doing with it’. Posters might help to raise awareness, intervention ‘champions’ is a good idea and could involve the therapists. Likes that the question card stands up and says it prompts you to look*. (Researcher field note, Physiotherapist interview)

### 3.3 Research aim 3: Identify modifiable areas for improvement in relation to usability, usefulness and acceptability of the intervention

Having identified the key messages to be conveyed in the booklet we condensed the information into four levels (five words; one sentence; one paragraph; two paragraphs; Table 2). These messages, as well as data gathered about patients' and staffs' capabilities, opportunities, and motivations to use the intervention guided proposed intervention changes. The intervention was revised with the aim of improving usability, usefulness, and acceptability. Implementation tools were developed to provide supportive prompts and minimize staff burden (Supporting Information: File S6, summary in Table 3). Changes were consulted with designers, the patient and public panel, and the programme management group.

**Table 2:** Intervention messages

Intervention message	Description
Central message: Active patient role	Patient to have an active role in preparing to be at home, by ‘doing’ (e.g., practising) and ‘knowing’ (e.g., questioning).
Secondary message: Upstream/downstream effects	Knowing that in hospital events have an effect at home, for example, poor preparedness to manage in hospital could lead to poor ability to manage at home.
Secondary message: Balancing risk	Acknowledging that there are risks and challenges to achieving an active patient role in the hospital, for both patients and staff, but the inability to address these risks in the hospital could mean they are transferred into the patient home.
Secondary message: System visibility	System processes are rarely articulated to patients, but by doing so we could help patients know what to expect next, respond appropriately and potentially save staff time.

**Table 3:** Intervention changes and implementation tools

Intervention changes	Implementation tools
Components and content were condensed to increase patients' capability to use the intervention.	Prompt cards to increase the ability of staff to introduce the intervention to patients and deliver key messages.
Core messages strengthened to increase patients' motivation to be more involved in their care.	Handout to promote awareness in staff and motivate them to use/interact with the intervention.
The mechanism for asking questions was made simpler and less ‘challenging’ to increase opportunities for patients to ask questions.	Visual prompts to raise staff awareness and increase opportunities for interacting with the intervention.
Patient‐friendly discharge letter was streamlined to reduce staff burden and increase motivation and ability for them to complete it.	Defined staff roles and activities to increase staff capability and promote opportunities for using/interacting with the intervention.
Alternative media were created to communicate core messages to increase the capability of patients to interact with the intervention.	Guidance for flexible local adaptation to increase ability and motivation for using the intervention.

## 4 DISCUSSION

In general staff, patients, and carers felt there was a need for, and understood the purpose of, the PACT intervention, thus it appears to have high coherence, which is a prerequisite for successful implementation.^40^ However, at an individual level, staff and patient willingness and ability to engage with the various intervention components varied. Among other things, the multi‐component nature of the intervention was burdensome and as anticipated, not all patients felt it relevant to them. A number of significant improvements to the PACT intervention were identified to help staff and patients further engage with the intervention. Before this study, we understood from the literature that hospital systems do not engender patient involvement in care^28^ and that from the patient's perspective involvement is not intuitive.^26^ From this we knew that to sufficiently alert patients to the importance of preparing for home while in hospital, some form of patient‐facing intervention was required. The key learning points from our study are that changing the culture of ‘non‐involvement’ requires intervention with strong but sensitive messages that are deliverable with minimal staff burden. Our findings have raised a number of interesting and important issues for research and practice, and we will deal with these in turn.

### 4.1 Implications for transitional care theory and research

There is emerging evidence that patients and their families are part of a healthcare system's potential for adapting to changing conditions, often stepping in when care fails or is suboptimal.^23^, ^41^ Examples of this support include chasing medications and appointments, querying medications and side effects and presenting to health services when they judge their condition needs to be escalated.^23^, ^42^, ^43^, ^44^ Patients and families also ‘step in’ to maintain the safety of their care at points of transition between healthcare services and settings.^23^ Our intervention was specifically designed to support this previously less acknowledged role of patients and families in improving the safety of transitional care. Where previous transitional interventions have acknowledged this role, it has usually been limited to information giving and encouraging ‘self‐management’.^16^, ^18^ In recognizing that the ability of older adults to undertake activities in their homes is directly impacted by the degree to which they undertake these activities within the hospital, we have shifted the focus on the transitional intervention from one that is about *discharge* to one that is situated *predischarge*. This study indicates that this shift in focus—giving patients a role in, and responsibility for, maintaining their own safety—is a recognized need.

The need for clinical teams to prepare adequately for discharge is well established empirically.^45^ This preparation has hitherto largely included a handover to clinical and social care teams, and other agencies, with less focus on the preparation of the patient or family themselves, outside of the (often rushed) discharge conversation. Therefore, focusing our intervention on supporting older adults to effectively ‘practice for being back at home’ throughout the hospital stay, is more novel. However, the recent interest in transitional care interventions that embrace healthcare as a complex adaptive system,^46^ suggests an extension of the adaptive potential to include older adults and their families would not be unwelcome.

### 4.2 Implications for implementation science theory and research

Another key issue emerging from our study is the need for the implementation of the PACT intervention to be adaptable at the point of delivery. We designed this study with minimal implementation support, specifically to examine what might be needed in our subsequent feasibility trial. Indeed, the mixed uptake of the intervention in this study can to some extent be explained by having an incomplete implementation plan. Although staff wanted tools (e.g., laminated prompt cards and handouts) to support the delivery of the fixed intervention components, our findings suggest that prescribing rigid implementation processes to be followed faithfully is unlikely to be successful. Indeed, while flexible implementation might increase perceived intervention complexity (posing its own implementation challenges), variation in the way healthcare practitioners responded to the intervention in our study reinforces the case for allowing adaptation to occur across different contexts (e.g., which staff groups introduce the booklet, answer patient questions, support patients to ‘do more’ on the ward etc.). Further, as noted by Penney et al.^46^ in their recent systematic review of interventions to reduce readmissions, ‘interventions that had an adaptive element were more successful, whether it be through allowing local self‐organization among individuals in the system, or recognizing that implementation is an evolutionary process that requires change over time’.

There is an increasing recognition that to be effective, interventions need to be considered ‘events in systems’, where complexity represents as much about the implementation context as the intervention itself.^31^, ^32^, ^33^ In our study we tried to address this in two key ways. First, from the outset, we explored and understood the intervention within its target context—in this case, wards with a high proportion of older adults. Second, we explored and sought to develop implementation approaches and tools that support engagement with the broad ‘functions’ of the intervention, while being clear to not necessarily prescribe rigid ‘forms’ that achieve these functions.^32^, ^47^ Our study provides further support for the need for ‘hybrid’ interventions that can be adapted at the point of delivery, to achieve change in complex healthcare systems.

### 4.3 Implications for practice

In our study, ward managers with the trust of, and positive influence over, their teams were found to improve engagement with the intervention. Despite this, we still found variation in the way that healthcare professionals supported the intervention. Such variation understandably exists in a staff group that experiences limited time and changeable teams. Although much of this is outside the control of the research team, we anticipate that staff engagement will be aided in future iterations by outlining specific roles—that is, what staff need to do or achieve. Who or which professional groups fulfil these roles will vary to suit the context of their ward team and a subsequent feasibility trial will explore the effectiveness of this.^48^

The issue of staff having to balance risk against potential patient benefits does, however, represent a real and very challenging obstacle for implementing an intervention that seeks to better prepare older adults for discharge. Despite their best intentions, healthcare professionals can be wary of well‐intentioned practices inadvertently resulting in harm, for example, increased patient mobility in wards contributing to falls. However, if these risks are not addressed in the ward environment, they are simply ‘kicked down the line’, with older people facing potentially greater risk within their community‐dwelling due to compounding frailty, deconditioning, and disorientation. Put simply, hesitancy to ‘balance the risks’ in hospital, effectively reduces the risk for hospital staff but raises it for discharged older adults and their families.

In theory, the hospital could—and arguably should—be an environment in which staff can support patients to safely practice skills for home. However, it is difficult to see how hospitals could take on more of this risk given an already overstretched health service.^49^ Although increasing adoption of ‘discharge to assess’ models^50^ acknowledges that hospitals are not the safest place to assess patients' longer‐term care and support needs, hospital staff and management also need to understand risk, not as something to be managed within the discreet boundaries of service but rather as distributed across services, settings, time, and people—with the biggest risk to the older adults themselves.

While our study findings have allowed us to make a set of recommended changes to the PACT intervention to increase its acceptability, and a further set of recommendations regarding implementation, the current situation in healthcare services presents a whole range of challenges that are out of our control and not limited to this intervention.

### 4.4 Study limitations

This was a small qualitative study that aimed to generate learning to facilitate improvements in the intervention and inform the development of an implementation strategy. The study therefore necessitated participants who could read and speak English and while we hoped for more ethnic diversity in the sample, it did not happen. Further, the study was limited to two specialisms within a single hospital. These two factors may make the findings specific to the context of the patient group and wards involved. However, we have confidence that the findings apply more broadly, based on significant knowledge gained from two preceding studies of the patient and staff experience across many other National Health Service organizations^17^, ^18^ and the involvement of professional and patient stakeholders.

## 5 CONCLUSION

The PACT intervention offers a novel means for improving care transitions for older people leaving the hospital, by supporting patients to be involved in their care *during their hospital stay* to support them to *manage when they return home*. This qualitative formative evaluation suggests that while there are a number of important challenges for supporting the PACT intervention, it was found to be acceptable for many patients and staff, with necessary changes and staff‐facing implementation tools identified, which aim to increase its usability. The intervention will be further developed and tested in a subsequent feasibility trial.

## AUTHOR CONTRIBUTIONS

Rosemary Shannon contributed to the design of the work and to the acquisition, analysis and interpretation of the data. She drafted the report and approved the final version. She is accountable for all aspects of the work in ensuring that questions related to the accuracy or integrity of any part of the work are appropriately investigated and resolved. Ruth Baxter contributed to the design of the work and to the acquisition, analysis and interpretation of the data. She contributed to drafting the report and approved the final version. She is accountable for all aspects of the work in ensuring that questions related to the accuracy or integrity of any part of the work are appropriately investigated and resolved. Natasha Hardicre contributed to the design of the work and to the acquisition, analysis and interpretation of the data. She contributed to drafting the report and approved the final version. She is accountable for all aspects of the work in ensuring that questions related to the accuracy or integrity of any part of the work are appropriately investigated and resolved. Tom Mills contributed to the acquisition, analysis and interpretation of the data. He contributed to drafting the report and approved the final version. He is accountable for all aspects of the work in ensuring that questions related to the accuracy or integrity of any part of the work are appropriately investigated and resolved. Jenni Murray contributed to the design of the work and the interpretation of the data. She contributed to drafting the report and approved the final version. She is accountable for all aspects of the work in ensuring that questions related to the accuracy or integrity of any part of the work are appropriately investigated and resolved. Rebecca Lawton lead the conception and design of the work and contributed to the interpretation of the data. She contributed to drafting the report and approved the final version. She is accountable for all aspects of the work in ensuring that questions related to the accuracy or integrity of any part of the work are appropriately investigated and resolved. Jane O'Hara lead the conception and design of the work and contributed to the interpretation of the data. She lead in drafting the report and approved the final version. She is accountable for all aspects of the work in ensuring that questions related to the accuracy or integrity of any part of the work are appropriately investigated and resolved.

## CONFLICT OF INTEREST

The authors declare no conflict of interest.

## Supporting information

Supporting information.

Supporting information.

Supporting information.

Supporting information.

Supporting information.

Supporting information.

## Data Availability

The data that support the findings of this study are available from the corresponding author upon reasonable request. The data are not publicly available due to privacy or ethical restrictions.
