# Supplementary figures and images for: A qualitative formative evaluation of a patient facing intervention to improve care transitions for older people moving from hospital to home
Source: Health Expect. 2022 Sep 3;25(6):2796–806. doi: 10.1111/hex.13560 (PMC9700184; doi:10.1111/hex.13560)

## Supplementary File 1.

*Outline of patient data collection*


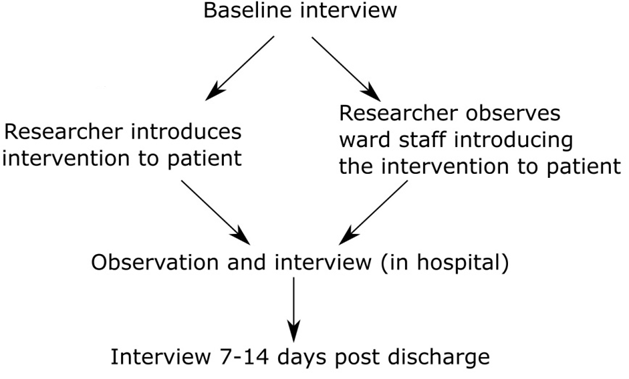

Supplement: Supplementary file 1 — Supporting information. [file HEX-25--s001.doc]
