# Supplementary material for: A qualitative formative evaluation of a patient facing intervention to improve care transitions for older people moving from hospital to home
Source: Health Expect. 2022 Sep 3;25(6):2796–806. doi: 10.1111/hex.13560 (PMC9700184; doi:10.1111/hex.13560)
Supplement: Supplementary file 2 — Supporting information. [file HEX-25--s003.doc]

**Supplementary file 2**

**Baseline topic guide for patients**

**Section 1: Being admitted to hospital**

1. Why have they been admitted to hospital? How did they get here?
2. How do they feel about being in hospital?
3. What do they think will happen next? What information have they been given? Do they feel they feel they have had had enough information?
4. How do they feel about going home?

**In relation to the questions above -**

1. How involved have they been / would they like to be in discussions about them and their treatment and care? How do they feel about this? Probe for: *choice*, **decision-making,** *information (given and received)*, **consultation about discharge process?**
2. Do they have any questions about their condition, treatment, or care? If so, have they asked anyone these questions?

**Section 2: find out about their living situation** e.g. who they live with, do they have any family, do they have any support at home etc.

**Section 3: Health and social issues**

- How are they managing their medication (getting them, taking them, understand them)?
- Have they fallen/problems with mobility?
- Pain (if so, well-managed)?
- How are they managing with normal daily activities (e.g. washing, dressing, going to the toilet)?
- Explore usual levels of independence

**Section 4: Perceptions of risks & concerns about the future**

1. What issues do they think they could face when they leave hospital to go home? Is anything been done about these?
2. Have they spoken to anybody about these things? If so, who?
