# Supplementary material for: A qualitative formative evaluation of a patient facing intervention to improve care transitions for older people moving from hospital to home
Source: Health Expect. 2022 Sep 3;25(6):2796–806. doi: 10.1111/hex.13560 (PMC9700184; doi:10.1111/hex.13560)
Supplement: Supplementary file 3 — Supporting information. [file HEX-25--s004.doc]

**Supplementary file 3**

**Follow-up interview topic guide for patients**

**Discharge:** Brief description of experience of discharge and care.

**The passport:**

What do you think the purpose of the passport is? What is it meant to support?

- Why do you think this is important?
- When staff introduced the passport to you, did you have any questions?

What challenges did you face once you were discharged from hospital?

- Did you know that you may face these challenges while you were in hospital, or is this something you’ve thought about since you’ve got home?
- What do you think could have been done while you were in hospital to prevent these things from happening?

**Perception of patient’s role:**

While you are in hospital, do you think patients have a role in supporting the care that they receive?

- What sorts of things are patients / should patients be responsible for while they are in hospital?
- In what way do you think you could play more of a role in your care?

While you were in hospital, is there anything you could have done to help your recovery / to help make things easier for being at home.

- Prompt Meds, ADLs, H&W, Esc – What could you have done? How would this help?
- How could staff have helped you with this?

**Booklet:**

What do you think this booklet aimed to do?

- How did you use it? How did that help you in hospital / at home?
- Which parts were useful / which were not? What stopped you from looking at it?
- What would entice you to read it?

Did you consider writing anything in the booklet?

- Do you think recording things would have been helpful? What stopped you?
- What would you have wanted to record?

**Question card:**

What do you think this question card was trying to support? How do you think patients could use it?

What sort of things did you want to talk to staff about while you were in hospital?

- Did you find the question card useful? Why / why not. Would you want to write those things on this question card? Do you think other patients would find it useful?
- Would you change anything? Why?

**Capability:**

- To what extent were you able to use the Passport while you were in hospital? What affected this?
- How did the Passport help you communicate with staff?
- Did the Passport prompt or encourage you to have these conversations?

**Opportunity:** (Prompt: environment, implementation, staff, family, researchers)

- What helped or encouraged you to use the PACT Passport?
- What discouraged you or stopped you from using the PACT Passport?
- How could staff have helped you?

**Motivation:**

- Did the Passport change *the way in which* you communicated with staff?
- Did the Passport made you *think differently* about being in hospital?
- Did the passport help you understand what to expect once you got home? e.g. symptoms, mobilising, tiredness, pain etc.

**Discharge letter:**

- What are your thoughts on this? How would it have helped?
- Explore acceptability of possible changes e.g. meds section (if appropriate)
