# Supplementary material for: A qualitative formative evaluation of a patient facing intervention to improve care transitions for older people moving from hospital to home
Source: Health Expect. 2022 Sep 3;25(6):2796–806. doi: 10.1111/hex.13560 (PMC9700184; doi:10.1111/hex.13560)
Supplement: Supplementary file 4 — Supporting information. [file HEX-25--s002.doc]

**Supplementary file 4**

**Topic guide: staff interviews**

In what ways do you think the passport aims to help patients?

- Why is this important?

Do you ever hear how patients are doing once they get home? How do you think they manage their Meds, H&W, ADLs, and Esc ?

- There is quite a lot of evidence now to suggest that patients struggle with x,x,x. What do you think may contribute to that?
- Do you think anything could be done in hospital to minimise these problems?
- Would you like to know what happens to some of your patients once they get home?

In what ways do you think patients can, or should, play more of a role in their care?

- What sorts of things could patients take some responsibility for?
- Why is this important?
- Prompt 4 functions

How do you currently support patients to:

- Understand their health condition? How do you sign post patients at discharge?
- Mobilise more on the ward? How does the risk of falling affect what you do? What are the implications of this? What happens if a patient falls?
- Understand their medications? What opportunities do you have to talk to patients about their medications? What are the barriers to this?
- Do some patients ever have the opportunity to practice taking their medications? What are the barriers to this? Do

What have you seen people on other wards do?

What would you like to improve, and what are the challenges to this?

**HCAs –** how do you currently draw out information from patients about things that are on their minds?

- What opportunities do you have to chat to patients during a shift?
- What helps you have these conversations?
- During these times, is there anything you could do to support patients to think about the things that are on their mind and to use the question card? What are the challenges of doing this?

**Passport Intro -**

How comfortable did you feel introducing the passport to patients? What worked well? What was difficult? How could we support you better?

WHY section - How much should we tell patients about the challenges or problems they may face when they go home?

- How do you feel having to talk to patients about these negative things?
- How could we support you to talk to patients about this? Give examples? – video / visual

When is a good time to introduce the passport to patients? How could this fit in with usual processes on the ward?

Did patients ever have any questions for you once you introduced it to them?

**Capability (psychological and physical):**

- Within your day to day role, to what extent were you able to interact with patients and carers as a result of them having the PACT Passport? (RQ4)
- How did the PACT Passport support you to involve patients and carers their care / to communicate with patients and carer? (RQ2)
- How does the PACT Passport prompt or empower you / prompt or empower patients or carers to have these conversations? (RQ2)

**Opportunity (physical and social environment):**

- What has helped you to use the PACT Passport? What has been a barrier to using the PACT Passport? (Prompt: environment, implementation) (RQ3 and 4)
- How have patients and carers prompted or supported you to interact with them more effectively / to use the PACT passport? (RQ2)

**Motivation (reflective and automatic mechanisms):**

- How has the PACT Passport changed *the way in which* you involve and communicate with patients or carers? (RQ2)
- Has the PACT Passport made you *think differently* about involving and communicating with patients and carers? (RQ2)

**General questions:**

- Which aspects of the PACT Passport work well and why? (RQ3 and 4)
- Which aspects of the PACT Passport could be improved and why? (RQ3 and 4)
