# Supplementary material for: A qualitative formative evaluation of a patient facing intervention to improve care transitions for older people moving from hospital to home
Source: Health Expect. 2022 Sep 3;25(6):2796–806. doi: 10.1111/hex.13560 (PMC9700184; doi:10.1111/hex.13560)
Supplement: Supplementary file 5 — Supporting information. [file HEX-25--s005.doc]

Contact type: (e.g. phone call, interview, observation session)

Location: (e.g. ‘ward’ or ‘patient home’)

Contact date & time:

Researcher initials:

Reason for contact:

People present: (non-identifiable e.g. staff role or participant ID number)

Field Notes

1. What were the main issues or themes that struck you regarding:

- How do patients and carers use the Passport? What do they think and feel about it?
- What do staff think and feel about the Passport, especially as it relates to patient involvement and communication?
- What changes need to be made to the PACT Passport to improve its usability and usefulness?
- How can the PACT Passport be effectively introduced to patients to maximise its use whilst minimising staff burden? How can staff be supported to engage with the Passport & patients?

1. Anything else that struck you as salient, interesting, illuminating or important in this contact?
2. Key questions or points to follow up at next contact (if applicable).
