# Supplementary material for: A qualitative formative evaluation of a patient facing intervention to improve care transitions for older people moving from hospital to home
Source: Health Expect. 2022 Sep 3;25(6):2796–806. doi: 10.1111/hex.13560 (PMC9700184; doi:10.1111/hex.13560)
Supplement: Supplementary file 6 — Supporting information. [file HEX-25--s006.doc]

## Supplementary File 6

#### Details of intervention changes

- Condensing - To increase engagement in this elderly population, the intervention needed to be more accessible and simpler. We condensed all intervention material down into a single item, reduced wording. While reducing the overall content and increasing accessibility, we needed to ensure that intervention retained authority and professionalism.
- Strengthening the message - Re-written to convey a stronger message encouraging patients to take an active role, added examples of what they can do, and what this could achieve. Renamed the title from to “Your care needs you!” to succinctly mirror this message and provide a directive to patients. Provided a clearer message regarding system navigation and escalation.
- Asking questions - Amended the question card to better support patients in raising things to discuss. Removed the word ‘question’ as this word is too strong or off-putting for most patients. Changed format to ‘cue’ cards which are used to signal that the patient has something to discuss. We explicitly linked the information given and suggested questions.
- Discharge letter - Created one shorter and one longer version of the patient-friendly discharge letter for wards to choose. Offered simple enhancements to existing discharge letters to reduce staff burden (e.g. pre-populated text to be copied into the current letter).
- Alternative media – Develop a short film to support booklet introduction and accessibility, and promote key messages. Create a simple website to hosts the video, booklet, and to signpost relatives to.

#### Details of implementation development

- Booklet introduction - Develop the prompt sheets, on small (A7) and larger (A4) card. To summarise three key points to convey: ‘what’ the intervention is, ‘why’ it is important or could help them, and ‘actions’ for the patient (read and discus it with visitors or staff).
- Staff awareness - Develop the ‘handout’ to guide ward staff in briefing their own teams about the intervention.
- Visual prompts – Develop posters addressed at patients, carers and staff (e.g. suggestions of how patients can be more active in hospital), to prompt or remind them of potential intervention benefits and how to use it.
- Staff roles - Propose specific which staff can take on to support intervention delivery. Primarily these are: a named person to introduce the intervention to patients; a person or small team to promote the intervention at ward level (‘coaches’). Further optional roles could be agreed locally and could include: eliciting and answering patient questions; completing the patient-friendly discharge letter; supporting specific patient activity (e.g. practising taking medications).
- Make it achievable for staff – 1. Communicate the potential intervention benefits as described by patient and staff themselves. 2. Make use of video, website and posters to limit staff burden (i.e. signpost patients and relatives to these). 3. Encourage the multidisciplinary team to share roles (and minimise nursing burden). 4. Emphasise flexibility and that activities can be graded to suit their ward and patient group.
